# Estimating Implementation and Operational Costs of an Integrated Tiered CD4 Service including Laboratory and Point of Care Testing in a Remote Health District in South Africa

**DOI:** 10.1371/journal.pone.0115420

**Published:** 2014-12-17

**Authors:** Naseem Cassim, Lindi M. Coetzee, Kathryn Schnippel, Deborah K. Glencross

**Affiliations:** 1 National Health Laboratory Service (NHLS), National Priority Programmes, Johannesburg, South Africa; 2 Department of Molecular Medicine and Haematology, Faculty of Health Sciences, University of Witwatersrand, Johannesburg, South Africa; 3 Health Economics and Epidemiology Research Office, Department of Clinical Medicine, Faculty of Health Sciences, University of Witwatersrand, Johannesburg, South Africa; Johns Hopkins Bloomberg School of Public Health, United States of America

## Abstract

**Background:**

An integrated tiered service delivery model (ITSDM) has been proposed to provide ‘full-coverage’ of CD4 services throughout South Africa. Five tiers are described, defined by testing volumes and number of referring health-facilities. These include: (1) Tier-1/decentralized point-of-care service (POC) in a single site; Tier-2/POC-hub servicing processing <30–40 samples from 8–10 health-clinics; Tier-3/Community laboratories servicing ∼50 health-clinics, processing <150 samples/day; high-volume centralized laboratories (Tier-4 and Tier-5) processing <300 or >600 samples/day and serving >100 or >200 health-clinics, respectively. The objective of this study was to establish costs of existing and ITSDM-tiers 1, 2 and 3 in a remote, under-serviced district in South Africa.

**Methods:**

Historical health-facility workload volumes from the Pixley-ka-Seme district, and the total volumes of CD4 tests performed by the adjacent district referral CD4 laboratories, linked to locations of all referring clinics and related laboratory-to-result turn-around time (LTR-TAT) data, were extracted from the NHLS Corporate-Data-Warehouse for the period April-2012 to March-2013. Tiers were costed separately (as a cost-per-result) including equipment, staffing, reagents and test consumable costs. A one-way sensitivity analyses provided for changes in reagent price, test volumes and personnel time.

**Results:**

The lowest cost-per-result was noted for the existing laboratory-based Tiers- 4 and 5 ($6.24 and $5.37 respectively), but with related increased LTR-TAT of >24–48 hours. Full service coverage with TAT <6-hours could be achieved with placement of twenty-seven Tier-1/POC or eight Tier-2/POC-hubs, at a cost-per-result of $32.32 and $15.88 respectively. A single district Tier-3 laboratory also ensured ‘full service coverage’ and <24 hour LTR-TAT for the district at $7.42 per-test.

**Conclusion:**

Implementing a single Tier-3/community laboratory to extend and improve delivery of services in Pixley-ka-Seme, with an estimated local ∼12–24-hour LTR-TAT, is ∼$2 more than existing referred services per-test, but 2–4 fold cheaper than implementing eight Tier-2/POC-hubs or providing twenty-seven Tier-1/POCT CD4 services.

## Introduction

The National Health Laboratory Service (NHLS) provides CD4 testing for staging HIV-infected patients and monitoring 2.3 million patients on antiretroviral therapy (ART) within the public health sector in South Africa. In 2013, the NHLS maintained an extensive network of 266 laboratories, of which 60/266 (22%) offered CD4 testing [1], serving around 3991 ART providing public health facilities in 52 health districts of South Africa [1].

Between April 2012 and March 2013, the NHLS/CD4 network provided 3.8 million CD4 tests [2]. CD4 testing is standardized across 60 laboratories, using Beckman Coulter equipment and the Pan*Leuco*Gating (PLG) method [3], [4]. The NHLS CD4 laboratories have a national expected within-laboratory turnaround time (TAT) of 24 hours and a national TAT target of laboratory-to-result (LTR) of 24–72 hours, irrespective of rural or urban areas serviced, to enable compliance with the National Department of Health (NDOH) treatment algorithm guidelines [5], where patients are required to return for their CD4 result after 7 days. For the reported period, the national median LTR-TAT was 55 hours (24–264 range, 95% confidence interval of 42–68 hours) [6]. While most of the CD4 capacitated laboratories are based within public sector hospitals in urban areas, many public health facilities in remote, rural districts need to refer samples in excess of 150 km to the nearest CD4 testing laboratory, potentially affecting specimen integrity and TAT of result delivery [7].

An integrated tiered service delivery model (ITSDM) for CD4 testing, described elsewhere [7], [8], has been proposed to ensure efficient, affordable and quality testing across South Africa and meet NDOH treatment algorithm requirements. This is a “full coverage” service model that aims at equitable access to CD4 testing services, irrespective of geographic location, by providing technology that appropriately matches service delivery requirements, placed in laboratories or in clinics. The model is dependent on volumes of tests (workload), the number of health-clinics served and distances from referring clinics to CD4 laboratories. In the ITSDM, POC technologies are used to deliver pathology services in remote areas where there is no reasonable access to a laboratory. The clinical impact of POC CD4 testing to improve patient enrolment onto treatment and retention in care, is described elsewhere [9]–[14] and beyond the scope of this study.

Six tiers of CD4 service delivery are defined in the ITSDM model. Briefly, (i) Tier-1 utilizes Point-of-Care (POC) technologies to provide a CD4 service in a single site (< five tests per system per day), and is reserved for hard-to-reach areas. Nursing staff, attending to patients, will likely operate the device and initiate patients onto ART. (ii) POC-Hubs (Tier-2), also known as ‘mini-laboratories’, serve 8–10 health-clinics and process <30–40 samples per day; exclusive use of POC equipment, placed in Tier-2 sites, will be operated by relatively unskilled laboratory technicians. (iii) Community laboratories (Tier-3) serve <50 referring health-clinics, processing <150 samples per day and use traditional but operator independent laboratory-based technology. (iv) District laboratories (Tier-4) process <350 samples per day whilst (v) high volume centralized ‘metro’ laboratories (Tier-5) serve >100–200 referring health-clinic facilities and process 350–1500 samples per day. Tier-6 [7] represents a national reference center to coordinate standardization, quality of testing, monitoring and evaluation across the national program and perform the function of coordinating ongoing training of staff.

The new South African National Health Insurance (NHI) model is being piloted in 11 districts [15]. The Pixley-ka-Seme district, a high priority for service delivery upgrade by the NDOH [15], is a remote district, situated in the sparsely populated Northern Cape province with a population of 86 351 and an HIV prevalence of 13.9% [16]. There are 44 public health facilities in this district, of which only 27 (61%) offer ART [17]. At the time of this analysis, there was no local CD4 servicing laboratory in Pixley-ka-Seme and CD4 tests from the district were referred to large Tier-4 or Tier-5 laboratories in adjacent districts. The district workload comprises a relatively small percentage (0.25%) of national test volumes and the recorded district LTR-TAT was noted to be 36–48 hours [6]. The Pixley-ka-Seme district therefore serves as a good case study for cost analysis of the ITSDM CD4 testing tiers, as the existing service is largely Tier-4 and Tier-5 based and lower tier ITSDM options could deliver better services with improved LTR-TAT in the area.

Initial estimates of the costs of the respective ITSDM tiers using data from the Pixley-ka-Seme district and surrounding provinces, lacked broader sensitivity analysis [18]. The purpose of this paper was to build on previously presented ITSDM costing analyses using an economic costing approach [14], and calculate the district CD4 costs and cost-per-result for existing, as well as proposed ITSDM service tiers, to improve service delivery in the district.

## Methods

Costing of CD4 services is multi-facetted and requires an in-depth assessment of several cost components to ensure that all related implementation and operational costs are reflected in the final cost of the test. Existing costing models [14], [19], [20] were adapted to estimate the total annual costs and cost-per-result per tier for tiers of the described ITSDM [7]. This Excel-based model uses historical volumes of tests and incurred expenditures or manufacturer-supplied pricing. A provider prospective is taken; all costs are reported for the NHLS as provider of CD4 testing. The main outcome of interest was the cost-per-test result (compared to the cost per patient or cost of CD4 reagents alone). A secondary outcome of turn-around-time was also considered. All costs are reported in local currency (ZAR) and in US$, using an exchange rate of ZAR9.26 per US$ [21]. All data used for this analysis was laboratory-based, aggregated facility data and therefore not considered human subject research. The Consolidated Health Economic Evaluation Reporting Standards (CHEERS) checklist was used in the preparation of the manuscript [22].

Lab-to-Result Turnaround-Time (LTR TAT) is defined as time from first registration onto the NHLS Laboratory (network) Information Management System (LIMS), to time of result authorization. Within-laboratory TAT refers to time from arrival in a testing laboratory to result authorization (TAT). Result authorization is a process within the laboratory at which a senior staff member releases results for printing and electronic access (SMS printer or web-based access). Following authorization, results are printed and delivered by an NHLS courier to each health facility or by mobile network short message service (SMS) printers in the originating site. The health workers are then responsible for placing the results in the patient folder.

At the time of this analysis, the Pixley-ka-Seme CD4 tests were referred and split between one regional Tier-4 (Kimberly) and another Tier-5 laboratory (Bloemfontein), respectively located 243 and 365 km away (Table 1). Historical health-facility test volumes (workload) from the Pixley-ka-Seme district, as well as the total volumes of work performed by the district referral CD4 laboratories, mentioned above, were extracted from the NHLS Corporate Data Warehouse (CDW) for the period April 2012 to March 2013. The Global Positioning System (GPS) coordinates/locations of all Pixley-ka-Seme referring clinics and the respective district NHLS laboratories, together with the related turnaround-time (LTR TAT) data was visible and linked to workload volume. From this analysis, it was possible to predict the expected number of tests that were likely to be requested daily from any specific referring site/clinic within the Pixley-ka-Seme district. This latter information, together with historical LTR-TAT, was used to propose an ITSDM tier of service that would enable <24 hour service delivery (Table 1) at any given site. Additional site visits confirmed existing locations of health facilities in the district providing ART [23], as well as confirming the location of existing NHLS (small and community) laboratories in Pixley-ka-Seme also assisted with decisions about determining prospective testing sites tiers (as Tier-1, Tier-2 or Tier-3). It was assumed that all clinics worked a minimum of 20 days per month (and an 8-hour day).

**Table 1:** CD4 tiers in Pixley-ka-Seme.

	Proposed Service Tier	Proposed Service Tier	Proposed Service Tier	Existing Laboratory Service-Tier	Existing Laboratory Service-Tier
Laboratory tier	Tier-1	Tier-2	Tier-3	Tier 4	Tier-5
Tier description	Decentralised POC service in a health-clinic providing ART	POC-Hub providing all related testing for HIV treatment and monitoring	Community CD4 laboratory	District CD4 laboratory	Centralised/CD4 laboratory
Name/type of site	ART initiating health facilities	Sub-district health facilities	De Aar	Kimberley	Pelonomi
Radius of referrals	<10 km^2^	10–50 km^2^	50–250 km^2^	250–300 km^2^	300–400 km^2^
Proposed (existing) number of testing sites	27	8	1	(1)	(1)
Number of health facilities serviced	27	44	44	(171)	(344)
Annual CD4 workload of district		10,080 for all of Pixley-ka-Seme		43 458, (*5040)	152 778, (*5040)
Cost-per-result	$32.32	$15.88	$7.42	$6.24	$5,37
**Estimated** cost of proposed services (current costs)	**$325 786 or**	**$160 070 or**	**$74 794**	($31 450) §	($26 904) §
*Monthly CD4 testing workload/site	27×31	8×105	1×840	3,622	12,732
(daily)	(1.14)	(5.25)	(42)	(181.1)	(636.6)
Proposed (existing) CD4 platform	Alere_PIMA, BD_FACSPresto or equivalent	Alere_PIMA, BD_FACSPresto or equivalent	BC_Epics XL MCL, BC_AquiosCL, BD_FACSCount or equivalent	(BC_CellMek and BC_MPL, or equivalent)	(BC_CellMek and BC_MPL, or equivalent)
Expected/(existing) LTR-TAT in hours	1	1–12	<24	(24–48)	(>36–48)

ITSDM CD4 service for the Pixley-ka-Seme district includes Tiers 1–3 (proposed new tiers, which can be implemented in varying combinations to ensure ‘full service coverage’), and Tier-4 and Tier-5 (comprising existing service). Further breakdown of tier costs is included in the Results and Fig. 2.

* (Existing Pixley workload split between two existing higher tier laboratories, geographically closest to referring health-clinic site).

§ Total −$58 354. Abbreviations: BD, Becton Dickinson Biosciences. BC, Beckman Coulter International.

The ITSDM provides for various options of service delivery to ensure ‘full coverage’ with a < than 24 hour LTR TAT. In Pixley-ka-Seme, the existing system of referral to adjacent district higher tier laboratories has led to unacceptable LTR-TAT. Three options of ITSDM service delivery were therefore considered to improve the LTR-TAT (Table 1, lines 4 and 5). Options included implementing (i) Tier-1 sites at health-clinics in the district offering ART, or (ii) Tier-2 testing hubs (using POC technologies) in existing NHLS depots or sub-district health facilities, serving all 44 health-clinics in the district, or lastly, (iii) a local Tier-3 community laboratory using traditional laboratory testing systems and existing staff in the biggest local town (De Aar) and servicing all health-clinics across Pixley-ka-Seme. Specifically, for the purposes of this study and calculation of the cost-per-test for each of the proposed ITSDM Tier-1, 2 or 3 services, the assumption made that CD4 testing was *not* referred to the nearby Tier-4 or Tier-5 laboratory (as in the existing service) but retained within the district itself. In other words, analysis was restricted to the historical test volumes of Pixley-ka-Seme, including data from each of the 44 public health facilities offering HCT, 27 of which offered ART in the district).

### Equipment costs

The laboratory-based CD4 testing tiers (Tiers-3, -4 and -5) use standardized equipment with inclusive maintenance, all of which was included in the fixed price per test procured through a national tender and subsequent service level agreement between Beckman Coulter SA and the NHLS. One of two systems was placed in accordance with daily test volumes (MPL/CellMek or Epics XLMCL/TQPrep respectively). Additional laboratory equipment required for CD4 testing, (not provided for with the CD4 procurement), was assumed to be purchased and included in the cost-per-test (i.e. mixers, fridge, pipettes, balance, biohazard safety cabinet, computer and air-conditioners). Connectivity costs were not included for newly implemented (Tier-3) laboratories as these sites are able to access and utilize existing NHLS network information technology (IT) infrastructure. Connectivity costs were however included for POC tiers (Tier-1 and 2), as some of the existing NHLS/NDOH facilities earmarked for Tier-2 POC-hubs and the clinic Tier-1 sites, may not have adequate IT infrastructure. Annual equivalent costs were based on a 5-year instrument expected use and a discount rate of 4%.

For the proposed POC tiers, (Tier-1 and 2), it was assumed that these facilities would be fitted with an inclusive Alere PIMA system (analyzer, printer, bag, computer, connectivity). No other equipment was required for Tier-1. Additional equipment (not provided with the PIMA instrument) was only foreseen for Tier-2 where the recommendation is to use pipettes and EDTA blood to accommodate multiple tests being performed across multiple POC technologies [24].

### Reagents and consumable costs

For each tier, the total annual recurrent costs for reagents and consumables were calculated using historical annual test volumes and the cost of individual test kits (and where necessary additional equipment) as at June 2013. For the laboratory-based Tiers 3–5, the fixed, *inclusive* Pan*Leuco*Gated (PLG) test cost from Beckman Coulter was used (details above). Reagent and consumable costs for these tiers are identical as a standardized methodology is used throughout all laboratories, irrespective of number of tests performed (and is inclusive of all reagents, sample preparation and analysis equipment and quality control). Additional sundry costs, per result, for all tiers, included laboratory gloves, sharps containers, printer paper and cartridges for printers, while POC tiers (1 and 2) had an additional cost included for sample collection kits for capillary bleeding (sold separately from test kit). For all tiers, the cost of waste management, external quality control (EQA), data capture and reporting were excluded, deemed equal across all tiers.

### Staff costs

Staff costs for all tiers were calculated from the bottom up perspective and assuming that staff time, not allocated to CD4 testing, could be allocated to another activity. The current staff grading (which determines salary) at the laboratory-based tiers was used to assess personnel costs in laboratories (using NHLS mid-point salary scales). In-house workflow analyses at the laboratories were used to calculate the percentage time spent, per staff member, on CD4 testing (including daily quality control procedures). The cost of a registration clerk was also included for tiers 2–5 based on the percentage time spent to register CD4 samples on the Laboratory Information Management System (LIMS). Staff costs for Tier-2 were allocated according to the premise that the hub would be run by a NHLS-employed medical technician (using NHLS mid-point salary scales), while a staff nurse (grade 3) on NDOH public sector salary scale 2013 was used to determine personnel costs at Tier 1 [25]. For the POC tiers, the estimated percentage of time that staff may spend on CD4 testing was calculated using averaged median daily test volume (workload), estimated from historical tests across the region (i.e. 6 per day for Tier-2 vs. 2 per day for Tier-1). In other words, the time taken to do one test was multiplied by averaged median daily test volume of tests performed per day (bottom up method) and expressed as a percentage of full time equivalents (FTE). This included time for instrument startup and daily quality controls (40–60minutes/day), with 20–30 minutes per test to accommodate other POC tests (Tier-2) or HCT activities (Tier 1). Staffing costs excluded the cost of training, monitoring and support site visits by management (considered Tier-6 “umbrella” activities applicable across all tiers of service).

### Excluded costs

Costs above the facility level (e.g. management and overheads, buildings) were excluded, as the proportion allocation to CD4 testing, would differ according to existing structures. It is assumed that ‘buildings and infrastructure’ for all tiers comprise a portion of a room within a public health facility and therefore, especially when discounted across the estimated 50 years of life for public buildings, the same across all tiers. Costs associated with sample transport were also excluded because a transport vehicle makes a daily run to the site to collect other samples and as a result there is no change in baseline costs whether CD4 tests are performed at POC or laboratory tier. Costs related to instrument failure or downtime were not included and assumed zero across all tiers based on expected planning for disaster recovery and/or operational redundancy planning in place.

### Sensitivity Analyses

**Figure 1:**
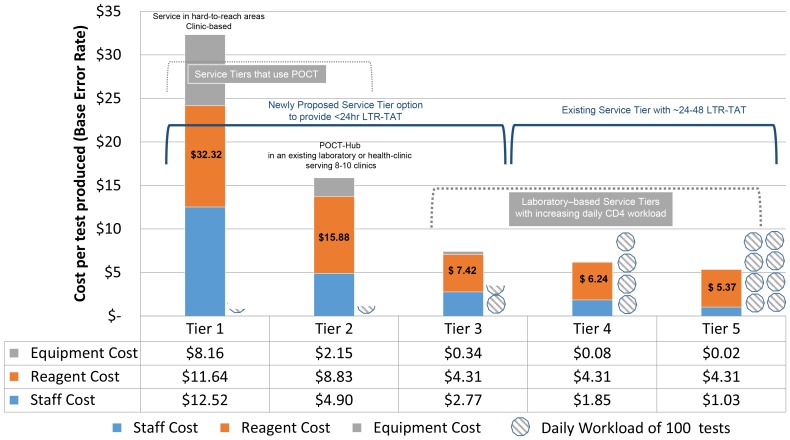
Breakdown of costs. Breakdown of individual cost components, i.e. equipment, reagent and staff costs, used to derive cost per test, at baseline error rates per tier (12% for Tier-1; 8% for Tier-2 and 1% for Tiers 3–5). Daily workload is graphically represented. Existing (Tier-4 and Tier-5) and proposed tiers (Tier-1, or Tier-2 or Tier-3) is shown, as well as service tiers that use POC technologies to CD4 deliver services.

One-way sensitivity analyses were performed to assess the impact of changing assumptions to the base model on annual costs and cost-per-result at each tier. Published error rates of 1% [26] were assumed for the laboratory Tiers (3–5) using flow cytometry platforms and included in unit costing (Fig. 1), but sensitivity analysis not performed due to established reliable performance [3], [26], [27].

Error rates were applied to POC-testing reagent and staff costs (where time spent could vary), performed in Tier-1 or Tier-2 sites. For Tier-1 and Tier-2, published error rates of 9-15% (mean 12%) and 6–10% (mean of 8%), were used respectively to assess impact on costs. In addition, for Tiers 1, 2 and 3 only, the impact of the volume of samples tested (workload) fluctuation on cost-per-result per tier was assessed by increasing, or decreasing test volumes by 25%. For Tiers 1 and 2, the impact of potential reduction in cartridge cost was assessed at minus 25% and minus 50%. Additionally, staffing time allocated for these tiers was reduced by 10 minutes from the baseline of 30 minutes for Tier-1, and 20 minutes for Tier-2. For Tier-2, it was assumed that staff could multitask while the POC CD4 instrument/s was analyzing samples.

## Results

### Proposals for new service sites

Historical health-facility test volumes extracted from CDW for the greater Pixley-ka-Seme district [5] is summarized in Table 1. These volumes were used to define daily testing volumes per tier/per testing facility from which the cost-per-result per tier was calculated. Twenty-seven health-clinics offering anti-retroviral treatment (ART) programs were identified as possible Tier-1 sites. In this option for ‘full coverage’ service, it is envisaged that the remaining 17 health clinics (of the 44 in the in the district offering only HIV Counseling and testing but not offering ART) would refer newly identified HIV positive patients for CD4 testing and ART enrolment, to their nearest ART-providing health-clinic. The second service option proposed included setting up eight POC-hubs/mini-laboratories in NHLS depots or sub-district health facilities, offering multiple POC services as described above. The existing sample courier network would ensure delivery of samples to POC-hubs. The last option was implementation of a Tier-3/Community laboratory established in the existing district center, De Aar, processing all the samples of district in one site and using the existing sample courier network that currently services all 44 health-clinics of the district.

### Cost breakdown per tier

#### Equipment costs

The cost of the flow cytometry and sample preparation equipment was included in the fixed reagent price per test, outlined below. The annual cost for laboratory and additional test-associated laboratory equipment required to establish and operate a laboratory CD4 service (Tiers 3–5) was $3,462 (R32 051). The annual costs for instruments and sundry equipment required for POC Tiers 1 and 2 were $21,654 (R200 449) and $60,354 (R558 696) respectively. Overall, annual equipment costs contributed <1US$ per result for laboratory Tiers (3–5) and between $2 and $8 per result for the POC Tiers 2 and 1 (Fig. 1).

#### Reagents and test consumable costs

The total annual reagents and test consumable costs for laboratory based Tier-3, Tier-4 and Tier-5 were $0,659 million (R6, 1 million), $0,19million (R1, 74 million) and $0, 43 million (R402 000) respectively. For Tier-2, the total annual reagent cost was $0.89 million (R824168), compared to $0.86 million (R796215) for Tier-1. This related to a contributing cost per result of $4.31 (R39.94) across the standardized laboratory testing platforms, while contributing $8.83 (R81.67) for Tier-2 and $11.64 (R107.71) for Tier-1 per test (Fig. 1).

#### Staffing costs

The total annual staff costs for laboratory Tiers 3, 4, and 5 were $27,879 (R258 073), $80,299 (R743 325) and $157526 (R1. 458 million) respectively. This related to a contributing staff cost per result of $2.77 (R25.60), $1.85 (R17.10) and $1.03 (R9.54), for Tiers 3–5 respectively (a decrease is evident with increasing automation used in higher service tiers, Fig. 1). For Tier-2/POC-hub, the cost of a medical technician spending 27% of their day on CD4 testing, was $49,343 (R456 770) per annum across eight hubs or $6,168 (R57 096) per POC hub per annum. This contributed a cost per result of $4.90 (R45.31). At Tier-1, a nursing staff member spending 12% of their day on CD4 testing, would cost $92,574 (R856 957) per annum across 27 sites, or $3,429 (R31 739) per POC site per annum, adding a cost per result of $12.52 (R115.93).

### Cost per test, per tier and the relationship to increasing workload and TAT

The overall cost per result for the laboratory-based tiers (Tiers 3–5) varied between $5.37 (R49.69) to $7.42 (R68.72) (Fig. 1). In comparison, the POC based tiers costs were between 2–4 times more per result than testing costs in a Tier-3 laboratory ($15.88 (R146.96, Tier-2, $32.32, R299.22, Tier-1). The incremental cost per result between Tier-3 vs. Tier-2 or Tier-1 was $8.45 (R78.24) and $24.90 (R230.50) respectively, whereas the incremental cost per result to perform a test in a more decentralised community laboratory (in comparison with a highly centralised laboratory was only $2.06 (R19.03) per test.

**Figure 2:**
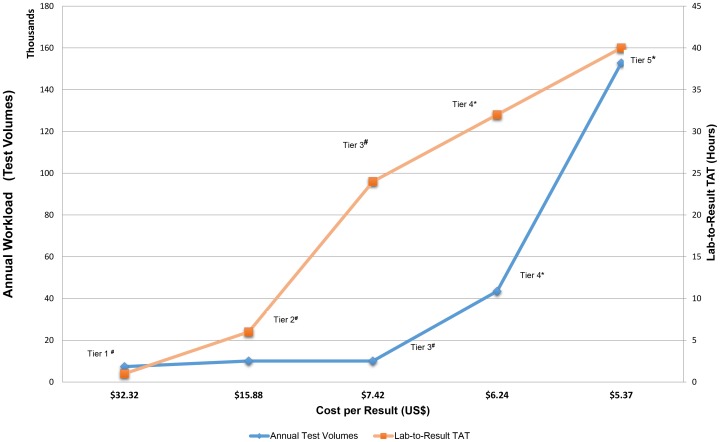
Relationship between costs, TAT and volumes. Comparison of expected laboratory-to-result turnaround-time (LTR-TAT, orange) and annual CD4 workload (test volumes, blue), per tier, versus cost-per-result (in US$). Tier 3, 4 and 5 laboratories with higher volumes have a lower cost but associated longer LTR-TAT, versus the POC tiers (Tiers 1 and 2) with fast TAT but cost 2–4 times more. Tier-3 emerges with the lowest cost despite lower workload but still meets <24-hour LTR-TAT and fulfils NDOH treatment algorithm requirements [5] where patients are requested to return for CD4 results at 7 days. (*Tiers 1, 2 and 3 are proposed services, #Tiers 4 and 5 are existing service tiers).

In a direct comparison of LTR-TAT versus annual test volumes and cost-per-result (Fig. 2), it is evident that lower cost per result is achieved at higher test volumes (Tier4 and Tier-5), or at a relatively decentralized laboratory (Tier-3), but with a related increase in TAT (24–48 hours). Providing same day results (1–6 hours) with POC testing (Tiers 1 and 2), increased the cost per result two to four-fold.

### Sensitivity analyses

**Figure 3:**
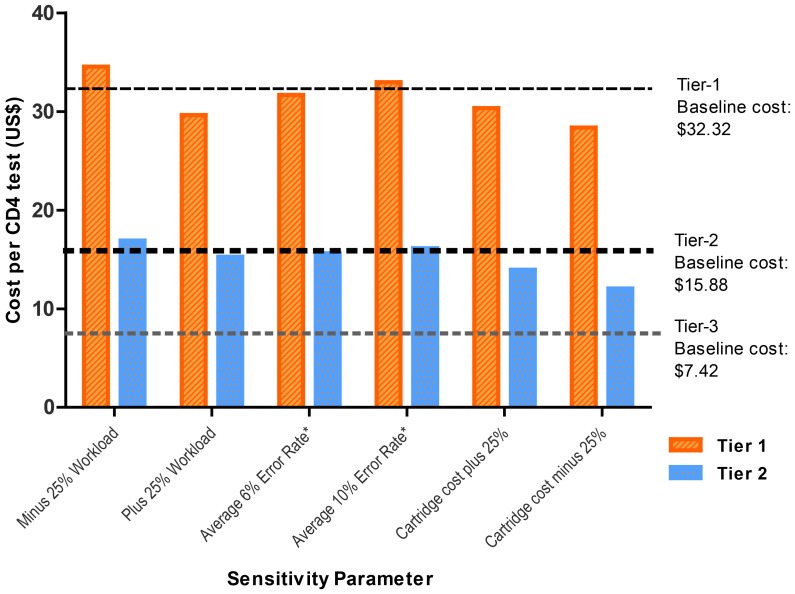
Sensitivity analysis. Sensitivity analysis for POC tiers indicating the impact of test volume, error rates and cartridge costs on cost-per-result. (High error rates of 10 and 15% for Tiers 2 and 1 respectively and low error rates of 6 and 9% per POC tier were used). Baseline cost for Tier-1 (upper dotted line), Tier-2 (lower dotted line) and Tier-3 (feint dotted line) is displayed for reference. This analysis confirms that POC cost is dependent upon volume of samples across a national programme and individual cost of cartridges.

Increasing or decreasing volumes by 25% marginally changed cost per result for Tier-3 ($6.80–8.46 vs. base case if $7.42). Similarly, for the POC hub (Tier-2) marginal changes were noted in costs with changes in test volumes ($15.26–16.91 vs. base case $15.88). For decentralized POC (Tier-1) the difference in cost/result was <$3.00. Incremental cost per result between Tiers 3 and 2 was not affected by changes in test volumes and remained at $8.45. In contrast, the incremental cost between Tier-3 and 1 was notably higher, between $22 (R203) to $26 (R241) (all Fig. 3).

As indicated in the Methods, different published error rates for POC testing were applied in the sensitivity analysis. The different error rates (6–10%) compared to base case of 8% resulted in a cost difference of $0.25 (R2.35). Error rates of 9 and 15% for Tier-1 resulted in a cost difference per result of $0.65 (R5.99), (also Fig. 3). Published error rate of 1% applied and added into laboratory CD4 costing, was negligible and changed costs by < than 5 US cents.

The sensitivity analysis also considered whether the procurement of a large volume of CD4 test cartridges would result in a drop in the price of the cartridges. For Tier-2, a 25% reduction in cartridge cost reduced the cost per result from $15.88 (R146.96) to $13.96 (R129.26), with a further decrease in cost at a 50% reduction in cartridge price to $12.05 (R111.57). For Tier-1, a 25% reduction in cartridge price reduced the cost per result of $30.34 (R281) vs. $28.36 (R263) at a 50% reduction rate (vs. $32.32–R299 base case).

Staffing time sensitivity analysis for Tier-1 indicated a reduction in cost per result from $32.32 to $29.91 ($2.41 reduction) compared to reduction of $1.98 for Tier-2. This is based on the reduction of the time allocated to POC CD4 testing by 10 minutes per sample.

**Figure 4:**
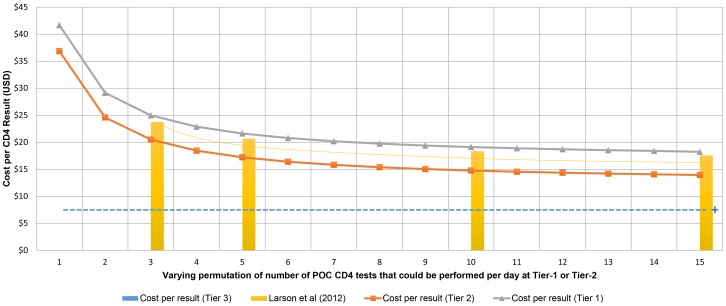
Costs per samples tested. Cost-per-result based on number of samples run per day for Tiers 1 and 2 (line graphs), compared to published POC data [14] (actual reported points as light yellow bar graphs with extrapolated curve) versus baseline cost-per-result for Tier-3 (pale blue dotted line). The ‘**+**’ at the end of line represents higher capacity of workload of Tier-3 services.

Additional sensitivity analysis was done to assess the change in cost per result of the POC tiers across a range of daily testing volumes based on the capacity of the POC instrument (n = 1–15). At daily testing volumes between 1 and 3 tests, the cost per result for both Tiers 1 and 2 were significantly higher than for volumes greater than 3 per day (>$24.97 vs. <$22.88). At maximum instrument capacity, the cost per result was $13.95 (R129.14) and $18.23 (R168.76) for Tiers 1 and 2 respectively. For both tiers, exceeding a daily volume of five samples/day, resulted in a plateau in cost per result (<$3.40 change from 5–15 samples/day). To put the POC costs into perspective, the cost per result for the community laboratory (Tier-3) with a capacity of up to 150 tests per day was shown to be lower (Fig. 4).

## Discussion

This study describes the unit costs of five tiers of the ITSDM, including service in high volume workload/centralized facilities through to POC service, in clinics, in hard-to-reach areas. There is no one-size-fits-all however, and the individual CD4 cost-per-test changes dramatically depending on how and where the test is performed. In the ITSDM, varying alternative service plans can be provided in any given district, by opting for a single tier approach, or a combination of one or more of the described tiers. For example, using Pixley-ka-Sema as a standard model to improve service-delivery, with a required < than 24-hour LTR-TAT, three alternative service plans are possible. (i) All Tier-1 POCT services in all twenty-seven ART-providing clinics; (ii) implementing local testing hubs (eight mini-labs that perform multiple HIV-related testing utilizing POC technologies in this study); or, lastly, (iii) referring all district CD4 testing to a local existing established general pathology laboratory, where CD4 testing can be introduced and a LTR-TAT of <24-hours maintained (in this study, the NHLS laboratory located in the main town, De Aar, was identified). A service plan with a combination of tiers may offer additional advantage i.e. a Tier-3 laboratory supplemented with Tier-1 sites in very remote or inaccessible areas of any given district will provide ‘full-coverage’.

The ITSDM costing model also has universal application and is generalizable; full costing can be performed to establish the cost of nationwide CD4 service for all health districts in South Africa [7], or other countries and programs, to find the best-fit for service, applying a combination of tiers that ensure optimal service delivery at least cost. Although CD4 testing was used in this costing exercise, the concept and detailed costing analysis described may also be applied to other laboratory tests where a spectrum of services, from centralized laboratories through to POC settings, is required, to deliver ‘full-coverage’ services.

The apportioning percentages of equipment, staffing and reagent costs were calculated (Fig. 1, Table 1) to enable an understanding of the relative cost contribution of individual components of a CD4 test, per result, within each service delivery tier. Between three laboratory Tiers, (Tier-3 to Tier-5), costs varied by 17%, the main contributing factor attributable to staffing costs. Test components, including the cost of major equipment (i.e. flow cytometers and sample preparation instruments) and reagents, did not contribute significantly to the differences of total cost-per-test noted across the laboratory tiers. The impact of standardized testing and related benefits of economies of scale in the laboratory tiers is evident here. This is largely attributable to the comprehensive all-inclusive cost per test provided by supplier, which includes all CD4 instruments and the maintenance thereof, quality control and reagents, provided through bulk procurement through prescribed national tendering processes. In countries outside of South Africa, the cost of equipment could however add significantly to laboratory cost-per-result. Additional laboratory equipment required (often already present in existing laboratories), including pipettes, blood mixer/vortex mixers, fridges, analytical scales, biohazard safety cabinets (Level 2), a computer and air-conditioners, etc. amongst others, added a only 0.4% to final cost per result. Minimal additional cost was associated with changing workload (increasing by 25%, or decreasing by 25%), contributing very marginally to the final cost-per-result across all laboratory tiers.

The convenience of providing a CD4 count at POC level (Tiers 1 and 2) made a CD4 test six times more expensive than providing CD4 testing from a centralized Tier-5 laboratory facility (this is the existing service plan for the district described here). This was mainly attributable to upfront implementation capital equipment costs that would be required across multiple sites, as well as higher ongoing reagent (up to 2.5 times) and staffing components (the latter contributing >70% for Tier-1 and Tier-2) required to provide ongoing near-patient services, in each POC site. Costs of POC testing and high ongoing costs were similar to those reported by Larson et al [14] (see Fig. 4). Costing analysis from this paper also indicated that providing decentralized POC (Tier-1) to secure service coverage is more than double the cost of providing POC CD4 in a mini-laboratory at Tier-2 hubs, or quadruple the cost of that in Tier-3 community laboratories. Sensitivity analyses confirmed that individual POC cost-per-result is also volume (workload) driven; fewer tests performed results performed in a single day, in a single site, results in a higher cost-per-result (see Fig. 4). As increasing number of tests are performed daily at any given site, the cost of daily controls is apportioned across all the samples tested that day. POC service costs even out at around five test samples per day; however, data from the district studied suggests that most Tier-1 POC sites would typically perform less than three samples per day (Fig. 4). Although reported as potentially problematic from a quality point of view [11]–[14], relatively high error rates (necessitating repeated testing) for POC testing increased cost-per-result by <1 UD$ per test. From a programmatic perspective, where hundreds of thousands of tests may be required, instrument or operator error at the POC could however add substantial additional cost and should not be ignored.

To date, higher near-patient testing costs documented here and elsewhere [14], have been largely covered by funding through non-governmental organisations [11], [28] or by research groups in South Africa [10], [13], [14], [28]–[33], frequently linked to studying the impact of use of POC devices in the context of improving linkage to HIV care. These reported costs are relatively high in comparison with equivalent laboratory testing. Bulk procurement, tendering processes and related economies of scale applied to more extensive use of POC CD4 testing, especially in the context of improved enrolment onto ART programs, could however facilitate better and sustainable pricing of up-front capital costs of equipment and reagents over the long term if the power of bulk procurement is taken advantage of. Although cost-per-result of a typical Tier 1 facility calculated in this study was generated in the context of delivery of nationwide pathology services, the costing is also relevant to use of POC CD4 in the context of strategies to improve newly diagnosed eligible HIV+ patient enrolment into care, specifically as attending nursing-personnel costs were included. It is especially important therefore to consider developing skills and capacity for laboratory services in regions where widespread use of POC is currently the only viable option to ensure CD4 service provision [13] and where resources may be limited.

The sensitivity analysis conducted to assess the impact of potential decrease PIMA cartridge prices on the overall cost per POC test, indicated a potential reduction of cost-per-result by between ∼$2.00 and ∼$4.00, with a reduction of total costs of 25 and 50% respectively. Overall, the sensitivity studies revealed that higher savings could be achieved by a combination of increased test volumes performed at each site and reduced PIMA^T^ cartridge prices. Reduction in staff time allocated for POC CD4 testing also showed a small impact on final cost-per-result of up to $2.41. It is therefore advisable that Tier-1 POC CD4 testing should be integrated into the activities of the HCT counselor/nursing staff; it is also envisaged that Tiers 2 staff will be employed solely by the NHLS to perform testing alongside other POC testing required for monitoring and treatment of ART.

The Tier-3 laboratory provided the best value for money and provided LTR-TAT in accordance with NDOH requirements. This study revealed that performing a CD4 service in the Tier-3-community laboratory cost less than one quarter of providing a CD4 test in a Tier-1-POC site, and just less than half of that to provide a CD4 service at a Tier-2/POC-hub facility. The total costs of providing CD4 testing in a Tier-3/community laboratory was $7.42 (R68.72). Although ∼$2 more expensive than a Tier-5 centralized laboratory CD4 (at $5.37), a CD4 test performed in a decentralized community laboratory is still substantially lower than the cost of $32.32 to provide a CD4 result at a POC level (Tiers 1 and 2). The main advantage of community laboratory decentralization is a service closer to the patient, with improvement of local TAT [34]. Although in some instances fully decentralized POC service may be the only solution to ensure a local service, the programmatic cost advantages of providing a service in a community laboratory (Tier-3) revealed in this study, should not be overlooked, especially where small laboratories already exist equipped to offer basic pathology services. Other pathology testing modules necessary for HIV work-up, e.g. GeneXpert, can be also added into these designated Tier-3 labs with existing amenities and staff, with minimal associated additional basic setup costs incurred.

The implementation of a tiered laboratory service across a national testing network provides for placement of CD4 laboratory testing modules in small community labs and additionally, utilizes of POC technologies in further remote sites to provide services in rural areas. Costs may be prohibitive if widespread POC services are proposed, with deployment of POC-technologies into each, and every ART providing clinic (see table 1 predicted costs of clinic-based Tier-1 services in the district studied). Spreading the national CD4 service load into smaller laboratories, as opposed to widespread use of POC services across multiple clinics, takes advantage of lower laboratory testing costs, providing a service that contains costs and still enables reaching communities in remote areas.

The analysis described in this paper was limited to a provider cost analysis of different tiers of CD4 service provision; results should be considered in the context and limitations thereof. The first limitation of any laboratory-based service is that results are not immediate; POC technologies were used in the ITSDM solely for extending pathology services in remote areas. The impact of use of POC technologies (like CD4 testing) in a HIV/AIDS programmatic context to improve newly diagnosed HIV+ patient's enrolment onto treatment and retention in care [10], [13], [14], [31] is an important from a perspective of retaining patients in care, but beyond the scope of this study. Another limitation is that the PIMA CD4 testing system was the only system costed. As new POC CD4 testing platforms (instrument and non-instrument based, [35]–[37]) become available for implementation into a national CD4 service, further review of costs will be required. The costs related to staff training, site support, providing an external quality assurance scheme, instrument failure and downtime as well as waste management were excluded across all tiers. However, as these systems and structures are currently part of laboratory-based testing facilities and not within clinics, inclusion of these costs would likely increase the relative cost of POC vs. laboratory test costs. Further, startup costs of rolling out new technology to POC sites as well as potential wastage of POC cartridges due to expiry, stock management, theft or inadequate handling (i.e. temperature control) was excluded from the analysis as there is insufficient information on which to model these costs. However, again, inclusion of these costs would likely increase the relative cost of POC versus laboratory test costs. While both clinics and laboratories, by statute, have systems for medical waste management, a cartridge-based CD4 testing system may put a greater strain on medical waste management than other types of CD4 test systems.

Another limitation of the study related to transportation costs. The associated costs and overheads of transporting samples were excluded as their marginal cost (or cost savings) were considered to be zero due to the need to transport other specimens from the clinic to the laboratory or from a decentralized laboratory to a centralized laboratory. However, over time, additional POC services to improve retention of HIV positive patients in care or to extend pathology services and changing needs of clinics and referral networks would alter transport needs and logistics, requiring a review of related costs.

## Conclusions

The ITSDM, described elsewhere, provides a framework for different levels and combinations of CD4 testing tiers to ensure ‘full coverage’ and <24 hour turn-around time (TAT) across a national program. Pixley-ka-Seme, a remotely situated, NHI (National Health Insurance) pilot district in South Africa, required upgrade of CD4 services to bring the district in line with the requirements of the National Department of Health HIV/AIDS treatment algorithm guidelines, which require a patient to return for their CD4 result at seven days. The TAT and district and referral workload were extracted and costs were calculated for the existing service (at a Tier-4 and a Tier-5 referral laboratory) with > than 24–48 hour TAT, as well as proposed lower ITSDM tiers aimed at <24-hour TAT (including Tier-1 POC, Tier-2 POC-Hub and Tier-3 decentralized/community service options). The analysis revealed that ‘full service coverage’ with <24-hour TAT could be achieved with either twenty-seven Tier-1/POC sites, eight Tier-2/POC-hubs or a single Tier-3 laboratory, at a cost-per-result of $32.32, $15.88 and $7.42 respectively. Existing referred services currently cost ∼$58 354 per year. Extending and improving CD4 services to provide ‘full-coverage’ to Pixley-ka-Sema could be achieved by implementing twenty-seven POC sites or eight POC-hubs, and cost 558% or 274% more per year respectively, but have potential for local improved patient enrolment onto ART programs. Establishing a single Tier-3 site in an existing community laboratory providing general pathology services, however, would cost an considerably less, an additional 28% per year more than the existing referred services, and provide the required <24-hours TAT that would be in line with current NDOH treatment guidelines. The outcomes of this study have implications for sustainability of national HIV/AIDS programs and should be carefully considered when making decisions about use of widespread POC services, national HIV/AIDS ART enrolment initiatives as well as possible budgetary constraints of a resource-limited national HIV/AIDS treatment programs.
